# Supervised Domain Adaptation for Automatic Sub-cortical Brain Structure Segmentation with Minimal User Interaction

**DOI:** 10.1038/s41598-019-43299-z

**Published:** 2019-05-01

**Authors:** Kaisar Kushibar, Sergi Valverde, Sandra González-Villà, Jose Bernal, Mariano Cabezas, Arnau Oliver, Xavier Lladó

**Affiliations:** Institute of Computer Vision and Robotics, University of Girona. Ed. P-IV, Campus Montilivi, University of Girona, 17003 Girona, Spain

**Keywords:** Brain imaging, Magnetic resonance imaging

## Abstract

In recent years, some convolutional neural networks (CNNs) have been proposed to segment sub-cortical brain structures from magnetic resonance images (MRIs). Although these methods provide accurate segmentation, there is a reproducibility issue regarding segmenting MRI volumes from different image domains – e.g., differences in protocol, scanner, and intensity profile. Thus, the network must be retrained from scratch to perform similarly in different imaging domains, limiting the applicability of such methods in clinical settings. In this paper, we employ the transfer learning strategy to solve the domain shift problem. We reduced the number of training images by leveraging the knowledge obtained by a pretrained network, and improved the training speed by reducing the number of trainable parameters of the CNN. We tested our method on two publicly available datasets – MICCAI 2012 and IBSR – and compared them with a commonly used approach: FIRST. Our method showed similar results to those obtained by a fully trained CNN, and our method used a remarkably smaller number of images from the target domain. Moreover, training the network with only one image from MICCAI 2012 and three images from IBSR datasets was sufficient to significantly outperform FIRST with (p < 0.001) and (p < 0.05), respectively.

## Introduction

Structural and morphological changes in brain structures are often associated with different neurodegenerative disorders such as bipolar disorder^1^, Alzheimer’s^2^, schizophrenia^3^, Parkinson’s disease^4^, and multiple sclerosis^5^. Many of these neurological abnormalities are usually diagnosed with careful analysis of the structural, T1-weighted (T1-w) magnetic resonance images (MRIs). Analysis of the sub-cortical structures – located beneath the cerebral cortex and including thalamus, caudate, putamen, pallidum, hippocampus, amygdala, and accumbens – is very important. Their deviations in volume over time are considered as biomarkers of the aforementioned diseases and are used for pre-operative evaluation and surgical planning^6^, longitudinal monitoring for disease progression or remission^7,8^.

Providing an accurate automated segmentation for the sub-cortical structures is very important because manually labelling an MRI volume is a time-consuming and tedious task^9^. Well-known, commonly used tools such as FIRST^10^ and FreeSurfer^11^ are available unsupervised methods. However, the advancement in computational technologies, such as graphics processing units (GPUs), has brought a new way to tackle the problem of image classification and segmentation using deep learning techniques, particularly, convolutional neural networks (CNNs). These approaches showed better results in many computer vision tasks such as image classification^12^, object recognition^13^ and segmentation^14^, than the traditional unsupervised methods that leverage hand-crafted features because the CNN features are learned directly from the training images^15^.

In recent years, deep learning has become a popular approach in medical imaging and brain MRI analysis^16,17^. Some methods based on deep learning strategies have also been proposed for brain structure segmentation^18–20^. The results of these approaches were promising; however, these methods were trained and tested in the same image domain – i.e., the same protocol, same MRI scanner, resolution and image contrast – and their behaviour with image domain change has not been evaluated. This is a common issue in supervised approaches, where a change in image domain causes an unexpected outcome because such methods learn a data distribution solely from the training set, making the supervised model less generalisable. Moreover, obtaining a well-trained deep CNN model requires a vast amount of training data with ground truth, which currently is a major issue in the medical image analysis field. Therefore, the traditional unsupervised methods – FIRST and FreeSurfer – are still the preferred tools of choice.

Few studies have proposed different methods to overcome the domain shift difficulty in medical images. These methods are often referred to as domain adaptation methods that tackle the problem of domain shift in medical images and address the broader statistical issue of out-of-sample generalisation in deep learning. One of the recent proposals includes domain adaptation using adversarial networks^21^. In this approach, a network contains an additional domain discriminator branch, which penalises the network when the features extracted from two different domains are distinct. In doing so, the network is forced to learn more domain invariant features. The loss computation with the discriminator does not require ground truth segmentation; therefore, adversarial domain adaptation could be carried out in an unsupervised manner for the target dataset. However, such approaches require a subtle parameter tuning and training of the network from scratch for different target domains. Another way to address the domain shift problem is via transfer learning, where the weights of an already trained network are fine tuned to adapt to a new target domain. This is inspired by the early convolutional layers capturing similar low-level features such as edges, curves and blobs. Accordingly, by performing an additional training on a smaller target dataset, it is possible to fine tune only some of the deep layers of the network that represent higher level features. Recent studies^22^ have shown that transfer learning and fine tuning decreases the training time drastically while demanding fewer training samples than that of full training. Additionally, the behaviour of the transfer learning strategy with different set of parameters has been recently analysed, indicating the effectiveness of this approach over full training for brain white matter hyperintensity segmentation^23^. In their work, the authors^23^ investigated two datasets containing fluid-attenuated inversion recovery (FLAIR) and magnetisation-prepared rapid gradient-echo (MPRAGE). The images of the two datasets were acquired with the same scanner and protocol, except for FLAIR images that had different image resolutions.

In this paper, we investigated the transfer learning and fine tuning strategies for domain adaptation on MRI volumes acquired with different scanners and protocols to segment sub-cortical brain structures. In our experiments, we employed a state-of-the-art deep learning based method that combines spatial and deep convolutional features for sub-cortical structure segmentation^19^. Within our study, we demonstrated the effect of domain shift on the neural network’s performance and analysed an adequate number of MRI volumes to adapt the CNN to a new domain and outperform traditional unsupervised methods. Because the sub-cortical structures drastically varied in their volumes, structure-wise performance after domain adaptation with different number of training images was also evaluated. Additionally, we performed an experiment to show the applicability of this study in real-case scenarios by accelerating the initial manual segmentation. Also, the training and testing time complexities were evaluated to examine how transfer learning could speed up the segmentation process compared with a fully trained neural network. Moreover, to encourage the reproducibility of our results, we made the source code used in training, transfer learning, Dice similarity coefficient (DSC), and statistical test calculations publicly available. Additionally, the manually corrected segmentation masks used in our experiments and label mappings for the MICCAI 2012 and IBSR datasets were made available for the community at https://github.com/NIC-VICOROB/sub-cortical_segmentation.

## Materials

### Datasets

In this work, we used two well-known, publicly available datasets – Internet Brain Segmentation Repository (IBSR) and MICCAI Multi-Atlas Labelling challenge (MICCAI 2012)^24^. More details on these datasets and their domain differences are provided in the following sections.

#### MICCAI 2012 dataset

The MICCAI 2012 dataset contains 35 images in total, which are split into 15 training and 20 testing image volumes according to the Multi-Atlas Labelling challenge. The 20 testing images were always used only for testing purposes and were never included in the training or validation processes. All images have a 1 × 1 × 1 *mm*^3^ resolution and image size of 256 × 256 × 256. Additionally, all image volumes in this dataset were acquired using the same MRI scanner – SIEMENS (1.5T). They were provided with manually annotated ground truth masks for 134 structures. We extracted 14 classes corresponding to the seven sub-cortical structures with left and right parts each.

#### IBSR

The IBSR dataset consists of 18 images with and image size of 256 × 256 × 128 and three different resolutions: 0.84 × 0.84 × 1.5 *mm*^3^, 0.94 × 0.94 × 1.5 *mm*^3^ and 1 × 1 × 1.5 *mm*^3^. The subject volumes of the IBSR dataset were obtained using two different MRI scanners: GE (1.5T) and SIEMENS (1.5T). Manually segmented ground truths for 43 different structures are provided^25^, and we extracted the 14 labels corresponding to the sub-cortical structures for our experiments. The MR brain images in this dataset and their manual segmentations were provided by the Center for Morphometric Analysis at Massachusetts General Hospital. Additionally, this dataset is a part of the Child and Adolescent NeuroDevelopment Initiative^26^ (CANDI) and was provided under the Creative Commons: Attribute license^27^.

#### Domain comparison

**Figure 1:**
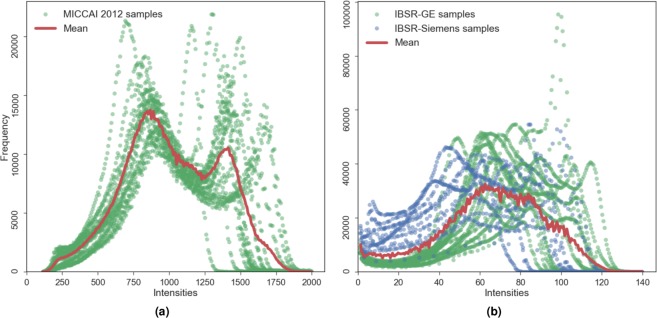
Intensity distributions in the brain area of the MRI volumes of MICCAI 2012 (**a**) and IBSR (**b**) datasets. The mean histogram is shown in solid red, and the intensity distributions of all images are shown in green for MICCAI 2012 and IBSR-GE datasets, and blue for IBSR-Siemens.

Proper selection of the datasets for the transfer learning experiments is crucial because the domain difference should be present to confirm the method’s robustness. As seen above in the selected datasets’ details, they differ in resolution and in MRI scanner type. The intensity distribution only in the brain area (i.e., skull-stripped) of these datasets also varies in terms of their profile (Fig. 1). The maximum intensity in the MICCAI 2012 volume reaches up to ≈2000 and that for the IBSR image is ≈140. This behaviour in intensity distribution is observed among the subjects in both datasets. Because the contrast and the intensity values of the structures of interest are represented from different distributions, pre-processing techniques such as contrast enhancement and histogram equalisation applied for each image individually cannot compensate for the imaging protocol differences between the datasets (e.g., image resolution). However, MRI volume standardisation across datasets using histogram matching could be an interesting line of research and is also analysed in this paper. Considering these variations, the datasets of interest perfectly fit the challenge of the domain shift problem.

Moreover, there is within-group variability of the intensity distributions of the subject volumes in the IBSR dataset among the three different image resolution groups due to the different MRI machines and various magnitudes of the partial volume effect. As shown in Fig. 1, the individual intensity distributions of the images in IBSR dataset also vary drastically, whereas the MRI volumes of MICCAI 2012 follow a similar profile. This intensity distribution variability in IBSR images makes the domain adaptation more challenging.

## Methods

### CNN architecture

**Figure 2:**
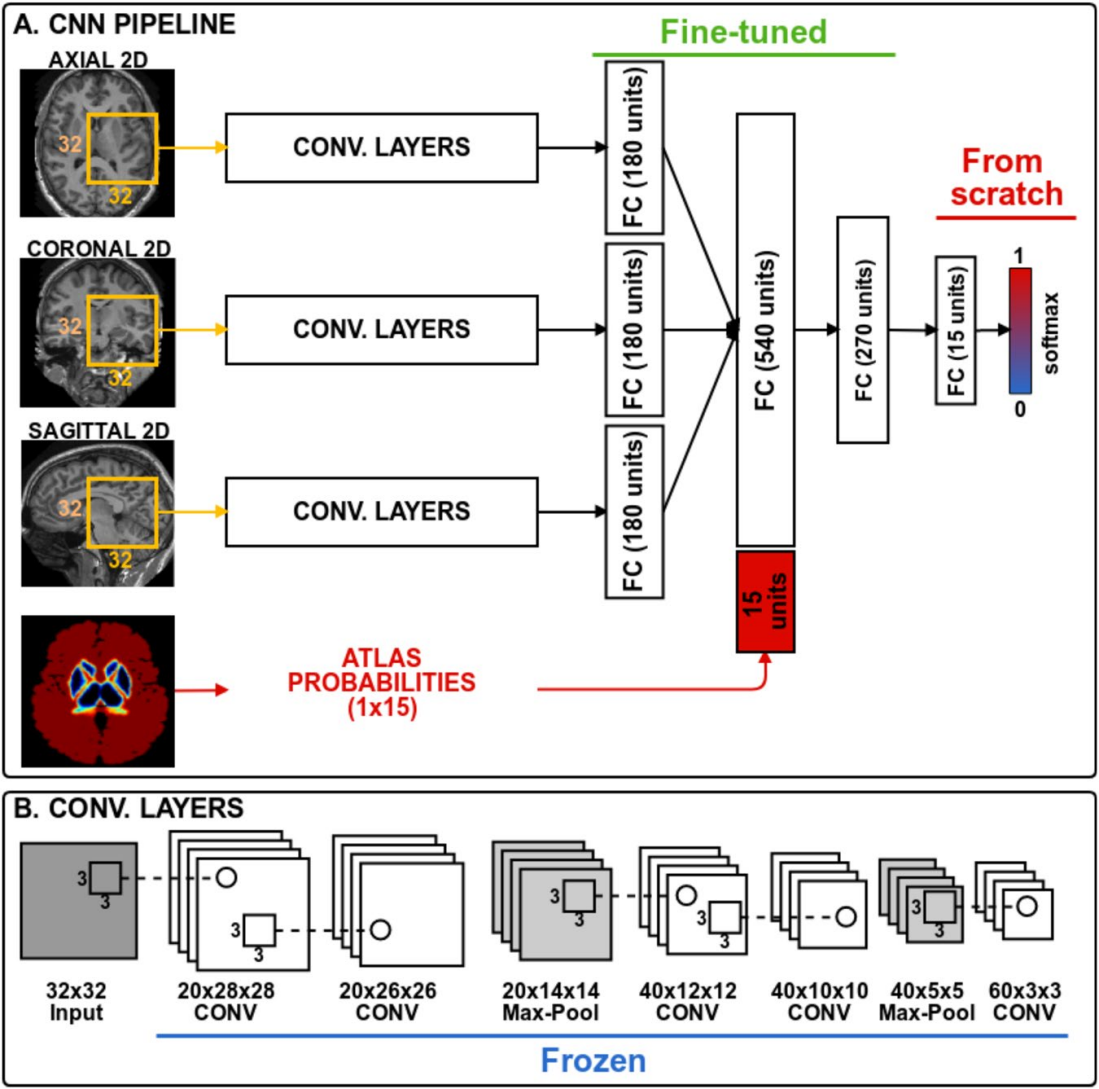
CNN architecture. The weights of all convolutional layers are frozen during transfer learning. The fully connected layers are fine-tuned and the last classification layer is trained from scratch.

The CNN architecture used in our experiments is shown in Fig. 2 and consists of three paths to process 2D patches of size 32 × 32. Each path is equipped with five convolutional layers, which are followed by a fully connected layer. The outputs of these paths are concatenated together with an additional 15 units corresponding to atlas probabilities. Finally, two fully connected layers are used to mine and classify the produced output by the preceding layers. Three 2D patches are extracted for every voxel from the axial, sagittal and coronal views of a 3D volume, making 2.5D patch samples. Next, each orthogonal 2D patch of the 2.5D sample is inputted into the three paths of the CNN. Although full 3D patches contain more surrounding information for a voxel, it is more memory demanding than using 2D patches. Therefore, employing 2.5D patches is a good trade-off between memory and contextual information for the network.

### Network training

To train the network, all samples were extracted from the 14 sub-cortical structures, and the background (negative) samples were selected only from the structure boundaries, which were obtained by dilating the ground truth by five voxels. Extracting the negative samples in this way allows the network to learn the most difficult areas of the region of interest that correspond to the structure borders^19^. Next, the atlas probabilities for 14 structures and the background are extracted, corresponding to all training samples and making a vector of size 15. These probabilities provide the network with spatial information and guide it to overcome intensity-based difficulties in some MRI volumes such as imaging artefacts and small tissue changes^19^. All the extracted samples were randomly split into training and validation sets with 75% and 25% proportions, respectively.

Once the training samples were extracted along with their atlas probabilities, the training of the network was performed in batches of 128 for 200 epochs. An early stopping policy was defined with patience 20 – i.e., the training stops if no increase was observed in the validation accuracy for 20 consecutive epochs. Optimisation was conducted for the categorical cross-entropy loss function using the Adam^28^ optimisation method with an initial learning rate of 10^−2^.

### Transfer learning

The transfer learning and fine tuning procedures were performed as follows. First, the network is fully trained from scratch as described above with one dataset, referred to as the source. Next, all the convolutional layers were frozen, and the weights of the last classification layer were reset. Accordingly, when the network is trained with the images from another dataset, referred to as a target, the weights of the convolutional layers are not updated. Additionally, the fully connected layers of the network were fine tuned – i.e., the weights were adjusted to better fit the new domain. We trained the softmax layer from scratch because it was used to classify the extracted features. Note that the initial learning rate was reduced to 10^−4^ during transfer learning to avoid rapid weight updates because most of the trainable parameters in the network were frozen.

### Network testing

To test a trained model, all 2.5D patches and corresponding atlas vectors were extracted from an MRI volume. Because the sub-cortical structures are located in the central part of the brain, the patches were obtained from a region of interest (ROI) defined by a mask from the dilated atlas probabilities. This helps to increase the processing speed and avoid false positives around the sub-cortical region. The network was well trained to classify the background only around the structures; therefore, some misclassified voxels may appear under the ROI. Those voxels were removed by keeping only the largest volume for each class.

### Image pre-processing

Before extracting patch samples from the MRI volumes to train the network, we performed some commonly used pre-processing steps:

- *Brain extraction* – i.e., removing non-brain structures, such as the eyes and skull, from an MRI volume. Compared with the previous study on sub-cortical structure segmentation^19^, we used the ROBEX (v1.2) tool^29^ instead of BET (fsl-v5.0)^30^. This is due to the robustness of the former method over the latter. Additionally, ROBEX does not require any parameter tuning compared with BET.
- *Atlas registration* – we performed non-linear registration of a template MRI with a probabilistic atlas to all images in the selected datasets. The probabilistic atlases were used in the network as explicit spatial information, which helps to improve the segmentation accuracy^19^. In this study, we used the well-known Harvard-Oxford probabilistic atlas^31^ distributed with the FSL tool (http://www.fmrib.ox.ac.uk/fsl). The non-linear registration of the atlas template to the subject volume was applied using the fast free-form deformation method^32^ that was implemented in the NiftyReg tool (http://cmictig.cs.ucl.ac.uk/wiki/index.php/NiftyReg).
- *Intensity normalisation* – as the maximum intensity values from both of the datasets differ drastically, all the subject volume intensities are normalised to have a zero mean and unit variance before training and testing the pipeline.

### Technical details

The network was implemented using the Keras^33^ deep learning library. The DSC scores were obtained using the Nipype^34^ data processing framework. The statistical tests were performed using the SciPy python package^35^. The Nibabel^36^ python package was used to read and write the medical imaging files.

### Experiments and evaluation

We trained our network with one dataset as the source and applied transfer learning with the other set as a target. Next, we repeated the same experiment but changed the datasets in the opposite order to show the method’s robustness. The target training MRI volumes, randomly chosen in previous iterations, are kept in the next iteration of transfer learning to have an unbiased estimate on changes in the results. For the sake of brevity, we compared our results with those of FIRST. According to our previous study^19^, FIRST showed better results than FreeSurfer to segment the sub-cortical structures in both selected datasets, and comparison of our method only with the former method is sufficient. The following experiments were carried out using the two selected datasets to evaluate the performance of domain adaptation using transfer learning.

- *From IBSR to MICCAI 2012* – All images from IBSR were used as the source, and domain adaptation was performed for the target MICCAI 2012 dataset. The results for the MICCAI 2012 dataset are shown for the 20 testing cases, and images for transfer learning were randomly selected from the 15 training cases.
- *From MICCAI 2012 to IBSR* – All images from MICCAI 2012 were used as the source, and the network was adapted to the target IBSR images. Because the IBSR dataset does not have training and testing splits, for each iteration of transfer learning, we randomly selected the corresponding number of MRI volumes and repeated the iteration with different images – e.g., a single iteration using one training image takes two steps: (1) training once with one image; (2) then training again with a different image to obtain an overall score for all 18 cases.
- *Grouping by MRI scanner* – The images of the IBSR dataset were split into two sets depending on the MRI scanner manufacturer for the IBSR-GE (12 images) and IBSR-Siemens (6 images) groups. The MICCAI 2012 dataset was used as the source, while the two new groups were set as targets. Due to the smaller number of images in the IBSR-Siemens group, transfer learning was performed for three iterations and the source dataset remained the same.
- *Corrected FIRST segmentation as ground truth* – Transfer learning was applied using the manually corrected segmentation outputs from FIRST as the ground truth. This shows how the initial domain adaptation of the network could be accelerated and avoid ground truth preparation from scratch.
  Additionally, the results of the first two experiments were also compared with two different approaches that could be used to tackle the domain shift problem:
- *Standardised (normalised) images* – The intensities of all images in both datasets were standardised to the mean histogram of the MICCAI 2012 using the two-stage method of Nyúl^37^. This experiment was performed to evaluate how intensity normalisation would affect the network’s performance because it has been shown to be effective for three classical image segmentation algorithms^38^.
- *Mixed datasets* – The network was trained from scratch using a mixed dataset containing normalised images of all subjects from source and iteratively added target volumes. This is to compare transfer learning with the performance of the network when there is more variability in the training data distribution.

Furthermore, the execution times regarding the neural network’s training and testing were evaluated to show how transfer learning can accelerate the convergence of the CNN.

We used the Dice similarity coefficient (DSC) to quantitatively analyse the results of our experiments. This metric evaluates the overlap of the automatic segmentation mask over the manually segmented ground truth. The DSC value varies between 0 (non-overlap) and 1 (full overlap). Because the larger structures contribute to the average DSC more than the smaller ones, penalised DSC by the inverse of the volume structure^39^ could be used to evaluate the performance of the method. In doing so, different segmentation approaches can be compared without volume bias. Therefore, we also used the weighted DSC to compare and verify the results of different experimental setups for consistency. Additionally, in our results, we showed the DSC values for each structure independently to demonstrate the evolution of the method’s performance along the iterations of transfer learning. Moreover, we used the pairwise non-parametric Wilcoxon signed-rank test (two-sided) to compare the statistical significance of our results with respect to the state-of-the-art tools. The results were considered significant for (*p* < 0.05).

## Results

### From IBSR to MICCAI 2012

In this section, the results for MICCAI 2012 are shown when the IBSR dataset was used as the source. Initially, the network was fully trained with all the images from IBSR, and transfer learning iterations were performed by incrementing the target training set’s size at a time. Table 1 summarises the DSC scores for FIRST, the results from full training, and the results when using transfer learning for the MICCAI 2012 dataset. Fine tuning the network with only one image drastically increased the performance of the network, significantly outperforming FIRST (*p* < 0.001). Incrementally adding an image volume to the target training set gradually improved the overall DSC score from 0.834 up to 0.860 (*p* < 0.001). Additionaly, it was possible to obtain a result similar to the fully trained network using only half of the training images. The domain shift effect on the trained network could be clearly seen from the results where no transfer learning was applied: an extreme performance drop compared with the fully trained network was observed in both, overall and structure-wise scores. These low scores were caused by confounding segmentation outputs of the network, where left and right parts for some structures were swapped.

**Table 1:** From IBSR to MICCAI 2012.

Str.	FIRST	FT	No TL	TL 1	TL 2	TL 3	TL 4	TL 5	TL 6	TL 7
Tha.L	0.889 ± 0.017	0.920 ± 0.017	0.529 ± 0.251	0.905 ± 0.014	0.904 ± 0.017	0.908 ± 0.017	0.909 ± 0.015	0.910 ± 0.015	0.912 ± 0.015	0.913 ± 0.015
Tha.R	0.890 ± 0.018	0.924 ± 0.016	0.418 ± 0.220	0.903 ± 0.012	0.908 ± 0.013	0.912 ± 0.011	0.914 ± 0.011	0.914 ± 0.012	0.912 ± 0.014	0.917 ± 0.012
Cau.L	0.797 ± 0.117	0.885 ± 0.071	0.694 ± 0.090	0.861 ± 0.066	0.867 ± 0.062	0.874 ± 0.063	0.875 ± 0.063	0.878 ± 0.061	0.880 ± 0.062	0.884 ± 0.060
Cau.R	0.837 ± 0.046	0.887 ± 0.057	0.774 ± 0.050	0.870 ± 0.049	0.874 ± 0.051	0.877 ± 0.050	0.883 ± 0.053	0.881 ± 0.053	0.886 ± 0.052	0.885 ± 0.053
Put.L	0.860 ± 0.080	0.909 ± 0.023	0.884 ± 0.023	0.903 ± 0.023	0.910 ± 0.024	0.911 ± 0.025	0.913 ± 0.023	0.914 ± 0.023	0.914 ± 0.023	0.915 ± 0.024
Put.R	0.876 ± 0.060	0.908 ± 0.031	0.884 ± 0.018	0.906 ± 0.024	0.910 ± 0.023	0.912 ± 0.023	0.913 ± 0.023	0.915 ± 0.024	0.913 ± 0.025	0.915 ± 0.024
Pal.L	0.815 ± 0.060	0.873 ± 0.101	0.374 ± 0.269	0.842 ± 0.032	0.856 ± 0.028	0.861 ± 0.028	0.862 ± 0.024	0.865 ± 0.023	0.866 ± 0.024	0.866 ± 0.024
Pal.R	0.799 ± 0.088	0.874 ± 0.049	0.111 ± 0.181	0.839 ± 0.043	0.850 ± 0.041	0.853 ± 0.043	0.857 ± 0.044	0.856 ± 0.048	0.858 ± 0.050	0.862 ± 0.045
Hip.L	0.809 ± 0.014	0.871 ± 0.020	0.808 ± 0.021	0.825 ± 0.034	0.835 ± 0.033	0.846 ± 0.026	0.849 ± 0.026	0.851 ± 0.024	0.854 ± 0.027	0.856 ± 0.026
Hip.R	0.810 ± 0.022	0.869 ± 0.020	0.822 ± 0.019	0.845 ± 0.019	0.841 ± 0.027	0.854 ± 0.020	0.854 ± 0.019	0.858 ± 0.020	0.861 ± 0.020	0.863 ± 0.020
Amy.L	0.721 ± 0.054	0.832 ± 0.032	0.669 ± 0.043	0.740 ± 0.043	0.777 ± 0.032	0.800 ± 0.031	0.809 ± 0.029	0.810 ± 0.030	0.812 ± 0.029	0.812 ± 0.033
Amy.R	0.707 ± 0.052	0.812 ± 0.027	0.613 ± 0.056	0.739 ± 0.047	0.750 ± 0.046	0.766 ± 0.044	0.774 ± 0.049	0.784 ± 0.040	0.788 ± 0.038	0.789 ± 0.042
Acc.L	0.699 ± 0.081	0.790 ± 0.052	0.693 ± 0.055	0.769 ± 0.050	0.779 ± 0.050	0.784 ± 0.055	0.788 ± 0.049	0.786 ± 0.050	0.789 ± 0.041	0.792 ± 0.046
Acc.R	0.678 ± 0.089	0.783 ± 0.067	0.238 ± 0.203	0.722 ± 0.093	0.729 ± 0.091	0.759 ± 0.091	0.763 ± 0.087	0.771 ± 0.090	0.774 ± 0.084	0.765 ± 0.084
Avg.	0.799 ± 0.094	0.867 ± 0.064	0.608 ± 0.272	0.834 ± 0.077	0.842 ± 0.073	0.851 ± 0.068	0.854 ± 0.066	0.857 ± 0.065	0.859 ± 0.063	0.860 ± 0.064
wAvg.	0.706 ± 0.061	0.803 ± 0.040	0.474 ± 0.083	0.754 ± 0.047	0.766 ± 0.046	0.783 ± 0.048	0.788 ± 0.046	0.791 ± 0.047	0.794 ± 0.042	0.792 ± 0.044

Mean ± standard deviation DSC values for FIRST, full training and transfer learning with an incremental number of training images. FT (Full training) – the network is trained from scratch with the MICCAI 2012 dataset. TL *X* – transfer learning with *X* number of target volumes. No TL – tested directly on the model trained with the IBSR dataset. The structure acronyms are as follows: left thalamus (Tha.L), right thalamus (Tha.R), left caudate (Cau.L), right caudate (Cau.R), left putamen (Put.L), right putamen (Put.R), left pallidum (Pal.L), right pallidum (Pal.R), left hippocampus (Hip.L), right hippocampus (Hip.R), left amygdala (Amy.L), right amygdala (Amy.R), left accumbens (Acc.L), right accumbens (Acc.R), average value (Avg.) and weighted average DSC (wAvg.).

**Figure 3:**
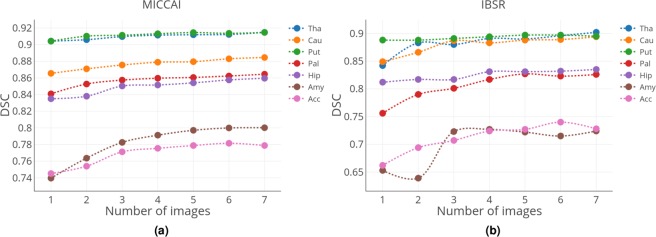
Change in the average DSC scores per structure with the increasing number of training images (left and right parts of all structures are averaged) for (**a**) MICCAI 2012 and (**b**) IBSR datasets as targets. Structure acronyms are thalamus (Tha), caudate (Cau), putamen (Put), pallidum (Pal), hippocampus (Hip), amygdala (Amy), and accumbens (Acc).

Improvement in the DSC values for each structure when increasing the number of target training images can be seen in Fig. 3(a). The highest DSC scores were achieved for the large structures such as the thalamus and putamen. Additionally, the difference in DSC when using one or seven images for training was not high, indicating that one image was adequate to obtain accurate segmentation for these structures. Interestingly, substantial improvement could be achieved for the smallest structures such as the amygdala and accumbens when the number of training images for transfer learning was increased.

**Figure 4:**
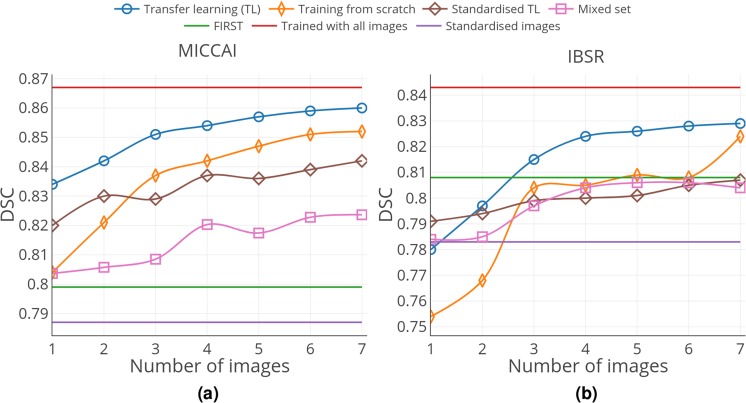
Overall average DSC results for (**a**) MICCAI 2012 and (**b**) IBSR datasets. The results are shown for seven iterations of transfer learning with original images, training from scratch, transfer learning with standardised images, and mixed set training. The horizontal lines correspond to the results of FIRST, full training, and the results of testing standardised images without transfer learning. The training volumes in each iteration for all cases are the same.

**Figure 5:**
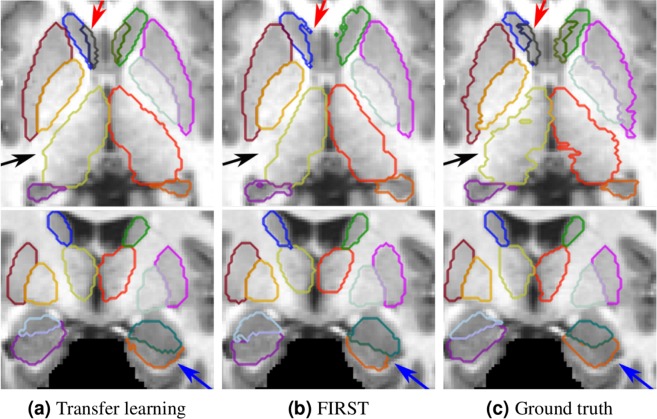
Qualitative results for the MICCAI 2012 dataset. (**a**) Transfer learning with one image; (**b**) segmentation result from FIRST; (**c**) ground truth. The results in the top row shown in axial and bottom ones shown in coronal views. The arrows indicate the following structures: red → accumbens, black → thalamus, blue → hippocampus.

Moreover, we observed that transfer learning actually helped to leverage previously acquired knowledge from the source dataset. Figure 4(a) illustrates the average DSC results for seven iterations of transfer learning with original images, training from scratch, transfer learning with standardised images, mixed set training, and the results of FIRST, full training, and the results of testing standardised images without transfer learning. The network performed worse in terms of the overall average DSC when trained from scratch with the same number of training images as that of transfer learning. Intensity normalisation definitely helped to improve the performance of the network when it was trained with the source and directly tested on the target without adapting the network to the new domain. As shown in Fig. 5(a), the standardised image segmentation was better, improving the average DSC from 0.608 to 0.787 for the MICCAI 2012 dataset (*p* < 0.001). However, no substantial improvement was observed when the standardised images were used for transfer learning. Additionally, using seven target training volumes was significantly less using the standardised images, with a DSC of 0.842 compared with 0.860 using the original images (*p* < 0.001). Additionally, transfer learning showed a better performance than that of the mixed dataset results. As illustrated in Fig. 4(a), the DSC values obtained by transfer learning was significantly higher for all iterations (*p* < 0.001). The results in the first three iterations were similar, slightly increasing from 0.806 to 0.808, and a significant increase starting from the fourth iteration, reaching 0.820 (*p* < 0.001). However, the performance did not improve any further. The average DSC values for each structure for the image standardisation and mixed set experiments are included in Supplementary Tables S1 and S3.

Some qualitative results for the MICCAI 2012 dataset are shown in Fig. 5, where transfer learning with a single image produced similar segmentation outputs to the ground truth. FIRST failed to properly segment the smallest structure, the accumbens (pointed with red arrow). Additionally, our method produced better segmentation for the thalamus structure (pointed with black arrow). Better segmentation using our method can also be observed for the hippocampus (pointed with blue arrow), where the curvature of the structure was preserved similar to the ground truth.

### From MICCAI 2012 to IBSR

For this experiment, we fully trained the network using all 15 training images from the MICCAI 2012 training dataset. Next, similar to the previous case, several transfer learning iterations were made using IBSR image volumes as the target and compared with the results of FIRST. Table 2 shows the obtained DSC values for FIRST, fully trained network with IBSR images using leave-one-out cross validation, and transfer learning results using zero to seven images. Very low DSC scores with high standard deviation in the results in No TL (see Table 2) showed the effect of the domain shift problem once again, confirming that this issue is present in both ways. The results were significantly improved when applying transfer learning with only one image, yielding a DSC value of 0.78 (*p* < 0.001). However, it was not higher than that of FIRST due to the different intensity distributions in MRI volumes of the IBSR dataset (Fig. 1). The results obtained by training the network with two images were similar to FIRST and not statistically significant (*p* > 0.05). A significant growth in average was observed when selecting three random images with DSC, reaching up to 0.815 compared with 0.808 with FIRST’s (*p* < 0.05). Increasing the number of target set images from four to seven resulted in similar DSC scores, slightly increasing from 0.824 to 0.829 (*p* > 0.05).

**Table 2:** From MICCAI 2012 to IBSR.

Str.	FIRST	LOO	No TL	TL 1	TL 2	TL 3	TL 4	TL 5	TL 6	TL 7
Tha.L	0.893 ± 0.017	0.910 ± 0.014	0.128 ± 0.226	0.847 ± 0.068	0.887 ± 0.017	0.882 ± 0.026	0.889 ± 0.024	0.890 ± 0.025	0.893 ± 0.016	0.898 ± 0.014
Tha.R	0.885 ± 0.012	0.914 ± 0.016	0.081 ± 0.173	0.837 ± 0.101	0.879 ± 0.031	0.877 ± 0.038	0.892 ± 0.030	0.890 ± 0.029	0.899 ± 0.015	0.906 ± 0.012
Cau.L	0.783 ± 0.044	0.896 ± 0.018	0.440 ± 0.290	0.857 ± 0.030	0.872 ± 0.031	0.890 ± 0.021	0.883 ± 0.022	0.887 ± 0.023	0.890 ± 0.025	0.894 ± 0.018
Cau.R	0.870 ± 0.027	0.896 ± 0.020	0.455 ± 0.306	0.840 ± 0.040	0.861 ± 0.034	0.883 ± 0.020	0.883 ± 0.020	0.889 ± 0.019	0.889 ± 0.021	0.895 ± 0.020
Put.L	0.869 ± 0.020	0.900 ± 0.014	0.845 ± 0.036	0.890 ± 0.028	0.889 ± 0.018	0.891 ± 0.022	0.896 ± 0.020	0.896 ± 0.022	0.895 ± 0.024	0.896 ± 0.020
Put.R	0.880 ± 0.010	0.904 ± 0.012	0.839 ± 0.029	0.886 ± 0.037	0.887 ± 0.025	0.890 ± 0.026	0.893 ± 0.027	0.897 ± 0.022	0.899 ± 0.020	0.894 ± 0.027
Pal.L	0.810 ± 0.033	0.825 ± 0.050	0.651 ± 0.141	0.737 ± 0.092	0.797 ± 0.034	0.801 ± 0.071	0.820 ± 0.033	0.830 ± 0.036	0.824 ± 0.041	0.826 ± 0.040
Pal.R	0.809 ± 0.037	0.829 ± 0.046	0.437 ± 0.243	0.775 ± 0.091	0.784 ± 0.039	0.800 ± 0.054	0.813 ± 0.030	0.824 ± 0.028	0.822 ± 0.029	0.825 ± 0.031
Hip.L	0.806 ± 0.023	0.851 ± 0.024	0.700 ± 0.050	0.811 ± 0.033	0.814 ± 0.031	0.819 ± 0.030	0.831 ± 0.032	0.829 ± 0.033	0.831 ± 0.030	0.834 ± 0.029
Hip.R	0.817 ± 0.023	0.851 ± 0.024	0.716 ± 0.040	0.813 ± 0.033	0.820 ± 0.028	0.814 ± 0.038	0.832 ± 0.029	0.833 ± 0.031	0.833 ± 0.027	0.836 ± 0.028
Amy.L	0.742 ± 0.064	0.763 ± 0.052	0.505 ± 0.147	0.647 ± 0.096	0.654 ± 0.062	0.735 ± 0.052	0.735 ± 0.043	0.729 ± 0.059	0.728 ± 0.052	0.738 ± 0.055
Amy.R	0.757 ± 0.062	0.768 ± 0.058	0.449 ± 0.126	0.659 ± 0.075	0.625 ± 0.075	0.711 ± 0.056	0.719 ± 0.056	0.716 ± 0.058	0.701 ± 0.072	0.711 ± 0.080
Acc.L	0.684 ± 0.098	0.744 ± 0.053	0.576 ± 0.113	0.668 ± 0.104	0.702 ± 0.117	0.710 ± 0.083	0.728 ± 0.070	0.734 ± 0.066	0.747 ± 0.080	0.722 ± 0.102
Acc.R	0.703 ± 0.076	0.752 ± 0.047	0.429 ± 0.107	0.656 ± 0.084	0.685 ± 0.099	0.704 ± 0.066	0.721 ± 0.068	0.720 ± 0.084	0.733 ± 0.063	0.733 ± 0.068
Avg.	0.808 ± 0.080	0.843 ± 0.071	0.518 ± 0.276	0.780 ± 0.111	0.797 ± 0.105	0.815 ± 0.085	0.824 ± 0.078	0.826 ± 0.081	0.828 ± 0.081	0.829 ± 0.085
wAvg.	0.714 ± 0.066	0.762 ± 0.037	0.505 ± 0.085	0.676 ± 0.051	0.704 ± 0.048	0.722 ± 0.047	0.738 ± 0.052	0.741 ± 0.053	0.750 ± 0.051	0.742 ± 0.052

Mean ± standard deviation DSC values for FIRST, full training and transfer learning with an incremental number of training images. LOO (Leave one out) – the results of leave one out cross validation. TL *X* – transfer learning with *X* number of target volumes. No TL – tested directly on the model trained with MICCAI 2012 dataset. The structure acronyms are as follows: left thalamus (Tha.L), right thalamus (Tha.R), left caudate (Cau.L), right caudate (Cau.R), left putamen (Put.L), right putamen (Put.R), left pallidum (Pal.L), right pallidum (Pal.R), left hippocampus (Hip.L), right hippocampus (Hip.R), left amygdala (Amy.L), right amygdala (Amy.R), left accumbens (Acc.L), right accumbens (Acc.R), average value (Avg.) and weighted average DSC (wAvg.).

The structure-wise improvement after each iteration of transfer learning for the IBSR dataset when using MICCAI 2012 as the source is shown in Fig. 3(b). Because the IBSR dataset comprises MRI volumes with different intensity distributions, we observed slight fluctuations in DSC for some structures. More substantial improvements were observed for the smallest sub-cortical structures, such as the pallidum, amygdala and accumbens, when the number of training images increased. By contrast, the larger structures were more accurately segmented starting from the first iteration of transfer learning and slightly increased through all iterations.

Similar to the previous experiment, we observed a benefit of using transfer learning over training from scratch. As shown in Fig. 4(b), transfer learning obtained better overall DSC than the network trained from scratch for all the iterations. Image standardisation was also useful in the case when no domain adaptation was applied. As shown in Fig. 4(b), using normalisation improved the average DSC from 0.518 to 0.783 (*p* < 0.001). However, similar to the case with the MICCAI 2012 dataset, the improvement throughout the iterations of transfer learning using the standardised images was not considerable. Additionally, the DSC for the standardised images using seven target training subjects (0.807) was significantly lower than that for the original images (0.829) with *p* < 0.001. The comparison of transfer learning and network trained from scratch using the mixed set of normalised images is shown in Fig. 4(b). In the first iteration, mixed set training showed a similar overall DSC of 0.784 to transfer learning (0.780), but the difference was not significant (*p* > 0.05). However, with more images, transfer learning was always significantly better than mixed set training (*p* < 0.001). In the case of mixed set training, the average DSC was significantly improved when adding more images, resulting in the highest DSC of 0.806 (five added images) (*p* < 0.001). However, it showed no further improvements when more images were added to the mixed set, instead reaching a plateau. The structure-wise results for the image standardisation and mixed set experiments are presented in Supplementary Tables S2 and S4.

**Figure 6:**
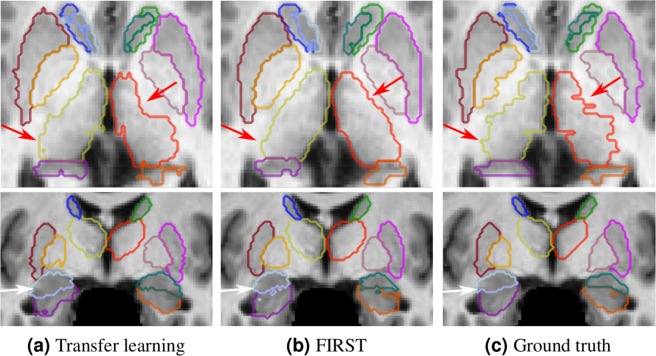
Qualitative results for the IBSR dataset. (**a**) Transfer learning with three images from three different intensity distribution groups; (**b**) segmentation result from FIRST; (**c**) ground truth. The results in the top row are shown in the axial view and those in the bottom are shown in the coronal views. The arrows indicate the following: red → left and right thalamus, white → amygdala and hippocampus structures.

Figure 6 illustrates some qualitative results obtained for this experiment. We showed the results of transfer learning using three training images because they were significantly better than those of FIRST (*p* < 0.05). As shown in the first row in Fig. 6(a), the segmentation result using transfer learning for the thalamus structure (indicated with red arrows) was more similar to the ground truth (Fig. 6c) than that of FIRST. Moreover, some spurious outputs could be observed in the boundaries of the adjacent amygdala and hippocampus structures (indicated with a white arrow) for the FIRST segmentation.

### Grouping by MRI scanner

Table 3 shows the results for the three iterations of transfer learning using the IBSR-GE group, the results of FIRST, training from scratch using leave-one-out cross-validation, and when no domain adaptation was applied. The average DSC of leave-one-out significantly outperformed FIRST (*p* < 0.001), whereas the transfer learning with two images yielded similar results, with an average DSC of 0.805 compared with 0.802 for FIRST. A significantly higher DSC of 0.814 (*p* < 0.05) could be achieved using only three images for domain adaptation than that of FIRST. The average DSC was very low when the images were tested directly on the network without domain adaptation, and applying transfer learning using one image improved the average DSC from 0.498 to 0.784; however, it was still lower than that of FIRST.

**Table 3:** From MICCAI 2012 to IBSR-GE.

Str.	FIRST	LOO	No TL	TL 1	TL 2	TL 3
Tha.L	0.894 ± 0.015	0.908 ± 0.014	0.092 ± 0.228	0.849 ± 0.039	0.873 ± 0.031	0.881 ± 0.031
Tha.R	0.882 ± 0.011	0.913 ± 0.017	0.122 ± 0.202	0.845 ± 0.081	0.881 ± 0.027	0.884 ± 0.028
Cau.L	0.771 ± 0.047	0.891 ± 0.017	0.329 ± 0.294	0.854 ± 0.039	0.867 ± 0.029	0.879 ± 0.020
Cau.R	0.806 ± 0.026	0.892 ± 0.022	0.351 ± 0.317	0.862 ± 0.034	0.882 ± 0.024	0.885 ± 0.026
Put.L	0.867 ± 0.023	0.896 ± 0.015	0.835 ± 0.038	0.875 ± 0.025	0.882 ± 0.018	0.887 ± 0.023
Put.R	0.883 ± 0.009	0.901 ± 0.012	0.831 ± 0.031	0.875 ± 0.031	0.883 ± 0.028	0.887 ± 0.024
Pal.L	0.802 ± 0.031	0.809 ± 0.052	0.652 ± 0.149	0.773 ± 0.074	0.798 ± 0.046	0.813 ± 0.033
Pal.R	0.809 ± 0.028	0.816 ± 0.049	0.491 ± 0.268	0.780 ± 0.070	0.782 ± 0.054	0.805 ± 0.047
Hip.L	0.804 ± 0.015	0.854 ± 0.021	0.693 ± 0.046	0.801 ± 0.034	0.812 ± 0.036	0.819 ± 0.031
Hip.R	0.812 ± 0.014	0.851 ± 0.022	0.711 ± 0.041	0.803 ± 0.03	0.814 ± 0.032	0.819 ± 0.037
Amy.L	0.745 ± 0.050	0.756 ± 0.045	0.464 ± 0.163	0.709 ± 0.046	0.719 ± 0.046	0.719 ± 0.062
Amy.R	0.758 ± 0.055	0.758 ± 0.058	0.443 ± 0.143	0.689 ± 0.068	0.736 ± 0.054	0.723 ± 0.059
Acc.L	0.655 ± 0.099	0.739 ± 0.059	0.563 ± 0.109	0.663 ± 0.099	0.669 ± 0.102	0.697 ± 0.065
Acc.R	0.691 ± 0.082	0.743 ± 0.048	0.392 ± 0.100	0.602 ± 0.080	0.673 ± 0.074	0.691 ± 0.061
Avg.	0.802 ± 0.083	0.838 ± 0.073	0.498 ± 0.284	0.784 ± 0.101	0.805 ± 0.089	0.814 ± 0.084
wAvg.	0.696 ± 0.069	0.754 ± 0.038	0.484 ± 0.078	0.657 ± 0.057	0.692 ± 0.063	0.712 ± 0.043

Mean ± standard deviation DSC values for FIRST, full training with leave-one-out cross-validation (LOO) and transfer learning with an incremental number of training images. TL *X* – transfer learning with *X* number of target volumes. S The structure acronyms are as follows: left thalamus (Tha.L), right thalamus (Tha.R), left caudate (Cau.L), right caudate (Cau.R), left putamen (Put.L), right putamen (Put.R), left pallidum (Pal.L), right pallidum (Pal.R), left hippocampus (Hip.L), right hippocampus (Hip.R), left amygdala (Amy.L), right amygdala (Amy.R), left accumbens (Acc.L), right accumbens (Acc.R), average value (Avg.) and weighted average DSC (wAvg.).

Table 4 shows the results for the IBSR-Siemens dataset using FIRST, training from scratch with leave-one-out cross-validation, no domain adaptation, and three iterations of transfer learning. The highest results were achieved using leave-one-out with an average DSC of 0.845 significantly outperforming FIRST at 0.818 (*p* < 0.001). The results of testing IBSR-Siemens images without transfer learning showed poor performance, as expected. The performance of the CNN was drastically improved using only one image for transfer learning, yielding a DSC of 0.826, slightly higher than that of FIRST but not statistically significant (*p* = 0.08). A significantly higher DSC than that of FIRST was obtained using two and three images for domain adaptation, reaching 0.840 and 0.846, respectively, with *p* < 0.001 for both cases. Moreover, the results of the third iteration of transfer learning were similar to the one of leave-one-out cross-validation with *p* = 0.87.

**Table 4:** From MICCAI 2012 to IBSR-Siemens.

Str.	FIRST	LOO	No TL	TL 1	TL 2	TL 3
Tha.L	0.892 ± 0.022	0.914 ± 0.013	0.201 ± 0.222	0.888 ± 0.019	0.891 ± 0.016	0.900 ± 0.014
Tha.R	0.889 ± 0.014	0.916 ± 0.014	0.000 ± 0.000	0.894 ± 0.015	0.902 ± 0.015	0.904 ± 0.015
Cau.L	0.805 ± 0.028	0.906 ± 0.017	0.663 ± 0.075	0.896 ± 0.021	0.905 ± 0.014	0.905 ± 0.015
Cau.R	0.892 ± 0.016	0.903 ± 0.015	0.663 ± 0.141	0.893 ± 0.014	0.892 ± 0.023	0.885 ± 0.026
Put.L	0.872 ± 0.016	0.909 ± 0.006	0.866 ± 0.021	0.901 ± 0.014	0.902 ± 0.007	0.910 ± 0.009
Put.R	0.875 ± 0.011	0.908 ± 0.010	0.856 ± 0.018	0.905 ± 0.013	0.907 ± 0.011	0.908 ± 0.013
Pal.L	0.827 ± 0.034	0.857 ± 0.028	0.649 ± 0.135	0.866 ± 0.020	0.852 ± 0.043	0.862 ± 0.027
Pal.R	0.808 ± 0.055	0.857 ± 0.024	0.328 ± 0.150	0.836 ± 0.017	0.835 ± 0.037	0.840 ± 0.034
Hip.L	0.811 ± 0.036	0.843 ± 0.030	0.715 ± 0.059	0.818 ± 0.028	0.821 ± 0.034	0.837 ± 0.025
Hip.R	0.826 ± 0.034	0.850 ± 0.031	0.725 ± 0.040	0.812 ± 0.025	0.827 ± 0.029	0.846 ± 0.024
Amy.L	0.736 ± 0.090	0.778 ± 0.067	0.587 ± 0.048	0.751 ± 0.064	0.787 ± 0.048	0.780 ± 0.060
Amy.R	0.756 ± 0.080	0.787 ± 0.057	0.463 ± 0.091	0.725 ± 0.059	0.733 ± 0.072	0.760 ± 0.052
Acc.L	0.742 ± 0.069	0.754 ± 0.041	0.603 ± 0.128	0.705 ± 0.052	0.754 ± 0.041	0.757 ± 0.040
Acc.R	0.725 ± 0.063	0.769 ± 0.044	0.502 ± 0.086	0.668 ± 0.045	0.749 ± 0.057	0.750 ± 0.039
Avg.	0.818 ± 0.073	0.854 ± 0.065	0.559 ± 0.256	0.826 ± 0.085	0.840 ± 0.070	0.846 ± 0.066
wAvg.	0.747 ± 0.044	0.778 ± 0.029	0.548 ± 0.082	0.713 ± 0.028	0.766 ± 0.036	0.769 ± 0.030

Mean ± standard deviation DSC values for FIRST, full training with leave-one-out cross-validation (LOO) and transfer learning with an incremental number of training images. TL *X* – transfer learning with *X* number of target volumes. The structure acronyms are as follows: left thalamus (Tha.L), right thalamus (Tha.R), left caudate (Cau.L), right caudate (Cau.R), left putamen (Put.L), right putamen (Put.R), left pallidum (Pal.L), right pallidum (Pal.R), left hippocampus (Hip.L), right hippocampus (Hip.R), left amygdala (Amy.L), right amygdala (Amy.R), left accumbens (Acc.L), right accumbens (Acc.R), average value (Avg.) and weighted average DSC (wAvg.).

### Corrected FIRST segmentation as ground truth

**Figure 7:**
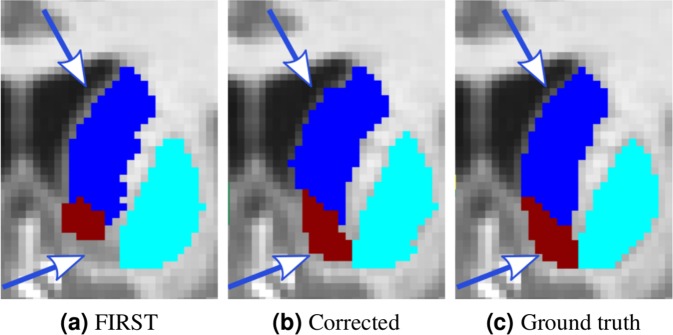
Corrected left accumbens and left caudate structures from the FIRST segmentation output. Coronal view: (**a**) FIRST segmentation; (**b**) corrected segmentation; (**c**) ground truth. Examples of some of the corrected areas are indicated with arrows.

Although the obtained results of the previous experiments are promising, we understand that manually segmenting all 14 sub-cortical structures, even for one image, is time consuming compared with, for instance, brain lesion segmentation^23^, which is a two-class problem. To overcome this issue, we also studied the use of the segmentation result of FIRST to train the network. FIRST provides a smooth unsupervised segmentation result; however, it does not perform well on small structures and structure boundaries. Therefore, in this experiment, we performed transfer learning using corrected FIRST segmentation outputs. The corrections included the following: removing outliers, filling holes and manually correcting some boundaries of the structures. We must note that the manual correction was performed by an operator who considered only the structures with good visual contrast from their surroundings. An example of a corrected segmentation for the left accumbens and left caudate structure boundaries is shown in Fig. 7. In this experiment, we used the IBSR dataset as the source, and two iterations of transfer learning with MICCAI 2012. The result when using one image with corrected labels for transfer learning was slightly higher than that of FIRST, yielding a DSC score of 0.805 ± 0.075. However, the difference was not statistically significant (*p* = 0.74). Significant improvements could be achieved when using two corrected images, obtaining a DSC of 0.817 ± 0.075 (*p* < 0.001). This shows that the output segmentation from FIRST could be used as a starting point for transfer learning and avoid manual segmentation of all 14 structures from scratch.

### Training and testing times

As shown in recent studies^22^, transfer learning allows the deep neural network to converge faster than a CNN trained from scratch. Our studies clearly confirmed this statement, by achieving much faster training time for the network. The average training time per epoch using a single training image was eight seconds, and it gradually increased when adding more images, reaching 63 seconds per epoch with seven training images. Comparison with full training, which took 832 seconds per epoch on average, the training time of transfer learning was less by two orders of magnitude when using one image and ten times less when using seven training images.

The testing time using our method was 1.3 minutes (run on GPU) + 3.7 minutes (atlas registration, run on CPU) per volume in average. On the other hand, FIRST took approximately 10 minutes to test one subject volume on average; however, this method does not require any training. All the experiments were run using a machine with a 3.40-GHz CPU clock and on a single TITAN-X GPU (NVIDIA corp, United States) with 12 GB of RAM memory.

## Discussion

Our experiments showed that the weights of the convolutional layers trained with the source dataset could generalise the features extracted from the target set. However, the domain shift problem requires a fine tuning of the way these features are interpreted. Thus, in our approach, we updated the weights of the fully connected layers and trained the classification layer from scratch.

In the experimental results, we showed that similar intensity distributions within the dataset helped the network to better generalise and provide a more predictable outcome. This was observed when the MICCAI 2012 dataset was used as the target, where we could see a smooth increase in the structure-wise DSC (Fig. 3a) and the overall average DSC (Table 1) after each iteration of transfer learning. By contrast, when the IBSR dataset was used as the target, the within-group variability of the MRI volumes in the dataset affected the results of transfer learning. As observed earlier (Table 2), substantial improvement in average DSC occurred in the first three iterations of transfer learning, but the results for the subsequent iterations reached a plateau. This means that the added images through the fourth and sevenths iterations did not provide the network with more useful information, making the learned weights of the CNN less general. The weights of the convolutional layers remained the same during transfer learning, indicating that they are not adapted to fit the new target domain. Therefore, when dealing with different intensity distributions, it is better to introduce more representative examples to make the fully connected layers better adapt to a new dataset. Additionally, one could notice that the results when using seven training images were not close to those using leave-one-out cross-validation. This behaviour was expected because in leave-one-out, 17 images were used to segment only one subject volume, which allowed the network to learn more variations present in this dataset. The within-group variability of the intensity distributions in the IBSR dataset showed unstable results for some structures during transfer learning (Fig. 3b). Additionally, random selection of the MRI volumes after every iteration added up to this behaviour because the outcome of the CNN relies on the descriptiveness of the training images. However, the overall trend showed an increase in DSC when more images were used for training.

**Figure 8:**
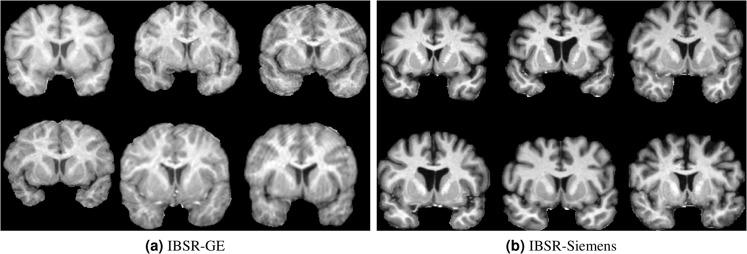
Illustration of some of the images from the (**a**) IBSR-GE and (**b**) IBSR-Siemens datasets.

Similar behaviour as in the first two experiments were observed when the images of the IBSR dataset were grouped into two sets by the MRI scanner type. As shown in Tables 3 and 4, for both new groups, transfer learning significantly outperformed FIRST using only three and two target training images for the IBSR-GE (*p* < 0.05) and IBSR-Siemens (*p* < 0.001) datasets, respectively. Ignoring the different number of images in both of these groups, it is interesting that the images of IBSR-GE were more difficult to segment for all considered segmentation approaches, including FIRST, deep learning, and domain adaptation. One cause may be the imaging artefacts present in some of the images from the IBSR-GE group. Figure 8 illustrates examples of the images from the two groups, indicating there are motion artefacts and more noise present in the IBSR-GE group than in the IBSR-Siemens images.

Comparison of transfer learning with training from scratch was also carried out to show the effectiveness of transferring knowledge. One could observe, for the case of IBSR (Fig. 4b), that the increase in DSC for transfer learning was smoother because the number of training images increased, whereas the results of the CNN trained from scratch showed a steep increase in the third iteration. This was due to the within-group variability of the image volumes in this dataset, and the selected image at this iteration was more representative than the previous two MRI scans. Additionally, because the training images for both were the same for each iteration, transfer learning compensated for the unseen cases and produced better results. A similar comparison (Fig. 4a) for the case of MICCAI 2012 dataset showed a gradual increase for both, transfer learning and training from scratch, with the former yielding better results in all iterations. Once more, we saw that a similarity in the intensity distributions within the target dataset makes a considerable difference.

**Figure 9:**
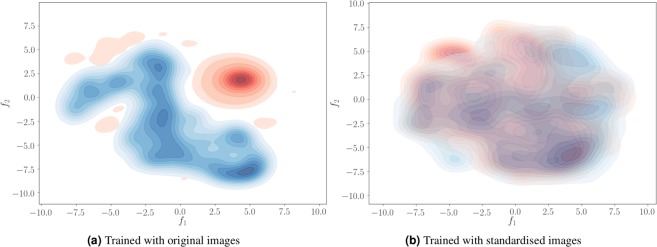
Visualisation of the concatenation layer outputs for the MICCAI 2012 (Red) and IBSR (Blue) datasets using original (**a**) and standardised images (**b**). 555-Dimensional feature vectors projected into 2-dimensional space using the t-SNE algorithm.

As shown in the results (Fig. 4), image standardisation for both datasets helped to achieve better results even without transfer learning. Interestingly, the outcome of transfer learning using one image with the original images was similar to that of normalised images without transfer learning. This means that transfer learning implicitly performs normalisation by adapting the weights of the fully connected layers to better interpret the features extracted by the convolutional layers. As shown in Fig. 9(a), the distributions of the feature vectors extracted from the concatenation layer for two datasets have a clear separation when the network is trained with the original images. By contrast, these distributions overlap when the network is trained with the standardised images (Fig. 9b). Accordingly, when no transfer learning is applied, the features extracted from the target images would be similar to the source, and the network produces better results even without initial training of the network. Although the image standardisation is helpful to directly use the network for segmenting images in other domains, it involves image intensity interpolation, which could be disadvantageous to obtain a better-adapted model using transfer learning.

Moreover, as shown in Fig. 4, training the network with a mixed set of images from different domains resulted in a slight increase in overall DSC when up to four images were added to the training set. However, there were no improvements observed with more images, but the average DSC stayed within a similar range. This behaviour of the network was caused by the interpolation in the IBSR image labels and the differences in the raters. According to these observations, domain adaptation using transfer learning would be a better choice than adding new images to the training process when there are only a few annotated images available in the target domain.

In the case of MICCAI 2012, all MRI volumes shared a similar intensity distribution, making the fully connected and classification layers of the network able well trained to overcome the results of FIRST using only one training image volume. On the other hand, the performance of the network also depends on the source dataset. This hypothesis arises due to the observation seen in the results with the IBSR dataset when MICCAI 2012 is used as the source. Because MICCAI 2012 images have a similar intensity profile, there are no filters in the convolutional layers that can consider discrepancies in intensity distribution. However, this assumption requires further analysis and experiments that should involve other datasets with similar within-dataset dissimilarities as in IBSR. This has not been analysed due to a lack of existing datasets that have ground truth segmentation labels for the sub-cortical structures. Therefore, we considered it a limitation of this study, and more elaboration is needed with more datasets containing intensity distribution dissimilarities to quantitatively analyse their impact on the performance of transfer learning.

The results of the weighted and unweighted DSC values had the same trend over the transfer learning iterations. This demonstrates that our method was consistent in terms of segmenting all the structures regardless their different sizes. However, subtle differences were observed where a higher DSC for one method was less in the weighted DSC. This shows that average DSC score favours high accuracy in the larger structures, whereas the impact of the smaller structures is lesser to the overall result. This issue could bring unreliable results in comparing different methods, especially, for evaluating sub-cortical structure segmentation approaches where the imbalance among classes is considerably large. Although the conventional DSC metric is mostly used in the literature, we encourage using the weighted DSC to verify the robustness of methods. The weighted average DSC values for all the experiments could be found in Supplementary Fig. S1.

As illustrated in the qualitative results (Figs 5 and 6), transfer learning produced segmentation masks that were more similar to the ground truth than FIRST. Because FIRST is based on the active shape model strategy, it tries to preserve the structure boundaries to the mean structure shape defined in the method itself. Therefore, some structural variations in shape may decrease the performance of this method. Moreover, FIRST found it difficult to properly define the boundaries for the adjacent amygdala and hippocampus structures (Fig. 6) due to their similar intensity profiles with no differentiable separation between them. By contrast, our method relies not only on local information but also on the surrounding context, considering the brain structural shape in the region.

One of the goals of transfer learning is to adapt a network that performs well in a new domain using only a few images for training. However, we have performed the domain adaptation using more target training images to confirm that the network does not overfit. According to the experimental results, adding more images did not further improve the results but showed similar performance in all the iterations of transfer learning. Therefore, in this paper, we have shown the results of only seven iterations where the network has reached the point of stability. The results of transfer learning with more than seven images is shown in Supplementary Table S5.

Although transfer learning was shown to be effective to deal with the domain adaptation problem, it still requires at least one manually annotated image volume, limiting our method to be used out of the box. However, the initial manual segmentation could be carried our more quickly by correcting the segmentation output from FIRST as shown earlier. Once the neural network is adapted, it could then be applied continuously with no retraining needed. Another limitation of our approach, as in all deep learning methods in general, is the necessity for the computational power of GPU. The training and testing times of such approaches will grow when run on CPU. Nonetheless, more powerful and affordable GPUs are becoming available.

## Conclusions

In this paper, we have demonstrated the application of transfer learning for sub-cortical structure segmentation to overcome the domain shift problem. In our experiments we have employed our previously proposed deep learning strategy that combines spatial and convolutional features. As shown in the results, we could achieve significantly better results than those of the well-known FIRST tool using one and three images for MICCAI 2012 (*p* < 0.001) and IBSR (*p* < 0.05) datasets, respectively. Accordingly, the transfer learning strategy is an excellent way to overcome the demand of deep learning methods for a large amount of data, especially in medical image analysis, where the ground truth availability is scarce. It allows us to use existing available datasets to bootstrap deep learning architectures and adapt the weights to fit to a new domain using much less training set than in full training.

We also showed that within-group variability in a dataset has an important effect on the network’s generalisability, suggesting that domain adaptation performs better when target images share a similar intensity distribution. Transferring the knowledge obtained from one dataset to another actually helped to achieve better performance. This was confirmed in our experiments, where transfer learning yielded superior results over the network trained from scratch using the same number of training images. Moreover, transfer learning was shown to be a better choice than other alternative solutions to the domain shift problem such as standardising images and mixed dataset training. Additionally, as we have seen in the experimental results, great acceleration in the training speed of the network could be achieved. Furthermore, we have made the source code of the pipeline available to the research community (https://github.com/NIC-VICOROB/sub-cortical_segmentation).

## Supplementary information

Supplementary materials
