# Supplementary material for: Supervised Domain Adaptation for Automatic Sub-cortical Brain Structure Segmentation with Minimal User Interaction
Source: Sci Rep. 2019 May 1;9:6742. doi: 10.1038/s41598-019-43299-z (PMC6494835; doi:10.1038/s41598-019-43299-z)
Supplement: Supplementary file 1 — Supplementary materials [file 41598_2019_43299_MOESM1_ESM.pdf]

Supervised Domain Adaptation for Automatic Sub-cortical Brain Structure Segmentation with Minimal User Interaction

Kaisar Kushibar[1,*], Sergi Valverde[1], Sandra González-Villà[1], Jose Bernal[1], Mariano Cabezas[1], Arnau Oliver[1], and Xavier Lladó[1]
[1]Institute of Computer Vision and Robotics, University of Girona. Ed. P-IV, Campus Montilivi, University of Girona, 17003 Girona (Spain).
*kaisar.kushibar@udg.edu

Supplementary Tables with structure-wise DSC and standard deviation values for experiments: transfer learning with standardised images; mixed set training from scratch; and transfer learning with more images.

Supplementary Table S1. From IBSR to MICCAI 2012 dataset. Average DSC and standard deviation results. **All images from both datasets are intensity normalised to the mean MICCAI 2012 histogram and transformed into MNI space.** TL X – transfer learning with X number of target volumes. No TL – tested directly on the model trained with MICCAI 2012 dataset. Structure acronyms are: left thalamus (Tha.L), right thalamus (Tha.R), left caudate (Cau.L), right caudate (Cau.R), left putamen (Put.L), right putamen (Put.R), left pallidum (Pal.L), right pallidum (Pal.R), left hippocampus (Hip.L), right hippocampus (Hip.R), left amygdala (Amy.L), right amygdala (Amy.R), left accumbens (Acc.L), right accumbens (Acc.R), average value (Avg.) and weighted average DSC (wAvg.)

# imgs	Tha.l	Tha.r	Cau.l	Cau.r	Put.l	Put.r	Pal.l	Pal.r	Hip.l	Hip.r	Amy.l	Amy.r	Acc.l	Acc.r	Avg.	wAvg
No TL	0.878	0.879	0.832	0.830	0.874	0.871	0.794	0.783	0.799	0.814	0.681	0.641	0.693	0.645	0.787	0.681
	0.011	0.010	0.066	0.046	0.022	0.022	0.045	0.049	0.024	0.018	0.055	0.051	0.051	0.073	0.094	0.038
TL 1	0.900	0.900	0.839	0.841	0.895	0.892	0.828	0.806	0.808	0.833	0.737	0.732	0.757	0.711	0.820	0.743
	0.013	0.012	0.063	0.046	0.021	0.022	0.029	0.055	0.025	0.018	0.041	0.043	0.046	0.082	0.075	0.040
TL 2	0.900	0.897	0.857	0.858	0.894	0.892	0.831	0.836	0.834	0.836	0.766	0.740	0.755	0.728	0.830	0.754
	0.016	0.013	0.062	0.042	0.020	0.020	0.030	0.047	0.023	0.017	0.031	0.033	0.053	0.075	0.069	0.040
TL 3	0.897	0.903	0.843	0.847	0.895	0.891	0.834	0.839	0.833	0.842	0.768	0.747	0.754	0.719	0.829	0.751
	0.015	0.013	0.070	0.053	0.023	0.023	0.025	0.044	0.024	0.019	0.037	0.049	0.054	0.087	0.072	0.047
TL 4	0.902	0.904	0.859	0.862	0.895	0.894	0.843	0.842	0.843	0.847	0.772	0.765	0.758	0.738	0.837	0.762
	0.018	0.015	0.065	0.050	0.023	0.021	0.027	0.043	0.022	0.019	0.026	0.036	0.054	0.071	0.067	0.039
TL 5	0.897	0.902	0.854	0.861	0.896	0.894	0.845	0.849	0.840	0.845	0.769	0.758	0.754	0.734	0.836	0.759
	0.014	0.012	0.066	0.049	0.022	0.022	0.028	0.042	0.020	0.017	0.039	0.041	0.053	0.079	0.069	0.041
TL 6	0.905	0.905	0.861	0.864	0.899	0.897	0.846	0.851	0.844	0.845	0.778	0.76	0.758	0.734	0.839	0.761
	0.016	0.012	0.07	0.05	0.023	0.022	0.03	0.04	0.019	0.018	0.033	0.037	0.056	0.072	0.069	0.041
TL 7	0.908	0.907	0.859	0.861	0.899	0.897	0.842	0.851	0.845	0.85	0.79	0.779	0.76	0.746	0.842	0.769
	0.015	0.012	0.067	0.05	0.022	0.024	0.031	0.043	0.018	0.016	0.029	0.037	0.055	0.076	0.066	0.042

Supplementary Table S2. From MICCAI 2012 to IBSR dataset. Average DSC and standard deviation results. **All images from both datasets are intensity normalised to the mean MICCAI 2012 histogram and transformed into MNI space.**. TL X – transfer learning with X number of target volumes. No TL – tested directly on the model trained with IBSR dataset. Structure acronyms are: left thalamus (Tha.L), right thalamus (Tha.R), left caudate (Cau.L), right caudate (Cau.R), left putamen (Put.L), right putamen (Put.R), left pallidum (Pal.L), right pallidum (Pal.R), left hippocampus (Hip.L), right hippocampus (Hip.R), left amygdala (Amy.L), right amygdala (Amy.R), left accumbens (Acc.L), right accumbens (Acc.R) and average value (Avg.)

# imgs	Tha.l	Tha.r	Cau.l	Cau.r	Put.l	Put.r	Pal.l	Pal.r	Hip.l	Hip.r	Amy.l	Amy.r	Acc.l	Acc.r	Avg.	wAvg.
No TL	0.861	0.866	0.842	0.847	0.877	0.867	0.775	0.787	0.799	0.801	0.679	0.648	0.690	0.617	0.783	0.671
	0.015	0.015	0.024	0.018	0.019	0.028	0.062	0.048	0.030	0.033	0.075	0.094	0.094	0.100	0.102	0.076
TL 1	0.878	0.881	0.840	0.838	0.866	0.862	0.774	0.781	0.796	0.800	0.733	0.722	0.654	0.642	0.791	0.673
	0.011	0.017	0.029	0.025	0.017	0.025	0.030	0.045	0.030	0.031	0.045	0.069	0.097	0.105	0.090	0.077
TL 2	0.876	0.879	0.845	0.843	0.866	0.860	0.780	0.784	0.799	0.799	0.713	0.708	0.692	0.675	0.794	0.700
	0.082	0.082	0.081	0.081	0.082	0.079	0.074	0.077	0.079	0.078	0.073	0.081	0.087	0.090	0.082	0.067
TL 3	0.869	0.870	0.848	0.851	0.875	0.871	0.790	0.779	0.806	0.801	0.720	0.707	0.696	0.699	0.799	0.712
	0.031	0.032	0.020	0.018	0.015	0.017	0.031	0.058	0.029	0.030	0.040	0.062	0.073	0.081	0.079	0.058
TL 4	0.879	0.880	0.856	0.855	0.874	0.869	0.787	0.765	0.804	0.806	0.714	0.696	0.708	0.698	0.800	0.715
	0.016	0.022	0.012	0.018	0.015	0.022	0.020	0.047	0.027	0.030	0.036	0.066	0.064	0.080	0.080	0.054
TL 5	0.880	0.883	0.855	0.858	0.873	0.871	0.788	0.784	0.807	0.810	0.708	0.689	0.706	0.702	0.801	0.716
	0.024	0.020	0.015	0.018	0.018	0.020	0.042	0.040	0.028	0.027	0.048	0.077	0.072	0.083	0.083	0.059
TL 6	0.890	0.889	0.861	0.858	0.873	0.874	0.787	0.790	0.812	0.811	0.725	0.703	0.697	0.696	0.805	0.712
	0.010	0.013	0.012	0.020	0.017	0.017	0.041	0.041	0.029	0.027	0.040	0.072	0.084	0.094	0.084	0.067
TL 7	0.892	0.891	0.863	0.868	0.880	0.877	0.788	0.792	0.818	0.816	0.732	0.688	0.690	0.710	0.807	0.715
	0.010	0.012	0.014	0.016	0.015	0.017	0.055	0.036	0.026	0.028	0.051	0.079	0.090	0.098	0.087	0.071

Supplementary Table S3. Average DSC and standard deviation for the MICCAI 2012 dataset with a network trained from scratch with a mix training set. **The mix set is composed of all IBSR images and iteratively added MICCAI 2012 images.** All images in both datasets were intensity normalised and transformed to the MNI space. MX X – mix set with X number of added target volumes. Structure acronyms are: left thalamus (Tha.L), right thalamus (Tha.R), left caudate (Cau.L), right caudate (Cau.R), left putamen (Put.L), right putamen (Put.R), left pallidum (Pal.L), right pallidum (Pal.R), left hippocampus (Hip.L), right hippocampus (Hip.R), left amygdala (Amy.L), right amygdala (Amy.R), left accumbens (Acc.L), right accumbens (Acc.R) and average value (Avg.)

# imgs	Tha.l	Tha.r	Cau.l	Cau.r	Put.l	Put.r	Pal.l	Pal.r	Hip.l	Hip.r	Amy.l	Amy.r	Acc.l	Acc.r	Avg.	wAvg.
MX 1	0.890	0.888	0.849	0.840	0.884	0.873	0.810	0.805	0.816	0.825	0.703	0.667	0.725	0.677	0.804	0.710
	0.015	0.012	0.064	0.048	0.020	0.023	0.032	0.051	0.016	0.016	0.045	0.051	0.045	0.078	0.086	0.042
MX 2	0.894	0.888	0.844	0.835	0.882	0.876	0.820	0.813	0.816	0.827	0.708	0.672	0.728	0.678	0.806	0.713
	0.015	0.016	0.063	0.048	0.022	0.023	0.042	0.050	0.025	0.020	0.053	0.073	0.046	0.081	0.087	0.046
MX 3	0.897	0.893	0.846	0.831	0.880	0.880	0.825	0.811	0.826	0.833	0.720	0.692	0.725	0.661	0.808	0.708
	0.017	0.016	0.068	0.048	0.020	0.024	0.031	0.055	0.024	0.020	0.049	0.065	0.057	0.087	0.088	0.055
MX 4	0.899	0.895	0.852	0.849	0.887	0.882	0.827	0.830	0.833	0.837	0.743	0.715	0.737	0.698	0.820	0.732
	0.016	0.013	0.063	0.043	0.025	0.024	0.043	0.041	0.019	0.016	0.036	0.054	0.044	0.074	0.077	0.041
MX 5	0.899	0.899	0.841	0.847	0.881	0.883	0.821	0.828	0.829	0.843	0.736	0.709	0.734	0.695	0.817	0.728
	0.017	0.013	0.071	0.048	0.022	0.024	0.035	0.048	0.026	0.017	0.058	0.067	0.055	0.088	0.082	0.049
MX 6	0.898	0.898	0.850	0.841	0.891	0.887	0.828	0.831	0.832	0.840	0.739	0.731	0.750	0.702	0.823	0.739
	0.017	0.013	0.073	0.051	0.023	0.023	0.040	0.052	0.026	0.015	0.050	0.050	0.054	0.090	0.079	0.051
MX 7	0.901	0.902	0.843	0.851	0.893	0.886	0.831	0.832	0.833	0.843	0.743	0.731	0.735	0.707	0.824	0.736
	0.017	0.014	0.070	0.047	0.021	0.022	0.041	0.047	0.026	0.019	0.062	0.056	0.060	0.090	0.080	0.054

Supplementary Table S4. Average DSC and standard deviation for the IBSR dataset with a network trained from scratch with a mix training set. **The mix set is composed of all MICCAI 2012 images and iteratively added IBSR images.** All images in both datasets were intensity normalised and transformed to the MNI space. MX X – mix set with X number of added target volumes. Structure acronyms are: left thalamus (Tha.L), right thalamus (Tha.R), left caudate (Cau.L), right caudate (Cau.R), left putamen (Put.L), right putamen (Put.R), left pallidum (Pal.L), right pallidum (Pal.R), left hippocampus (Hip.L), right hippocampus (Hip.R), left amygdala (Amy.L), right amygdala (Amy.R), left accumbens (Acc.L), right accumbens (Acc.R) and average value (Avg.)

# imgs	Tha.l	Tha.r	Cau.l	Cau.r	Put.l	Put.r	Pal.l	Pal.r	Hip.l	Hip.r	Amy.l	Amy.r	Acc.l	Acc.r	Avg.	wAvg.
MX 1	0.864	0.871	0.839	0.844	0.873	0.858	0.775	0.761	0.799	0.794	0.704	0.671	0.684	0.645	0.784	0.681
	0.014	0.013	0.025	0.022	0.019	0.033	0.061	0.063	0.027	0.033	0.041	0.073	0.083	0.092	0.091	0.068
MX 2	0.863	0.870	0.831	0.845	0.870	0.851	0.779	0.764	0.796	0.804	0.693	0.665	0.694	0.671	0.785	0.695
	0.011	0.011	0.035	0.017	0.019	0.034	0.058	0.059	0.032	0.034	0.051	0.095	0.088	0.096	0.091	0.072
MX 3	0.872	0.876	0.844	0.854	0.880	0.871	0.793	0.796	0.807	0.806	0.713	0.676	0.697	0.675	0.797	0.702
	0.014	0.014	0.029	0.018	0.013	0.020	0.036	0.036	0.028	0.031	0.053	0.078	0.083	0.089	0.087	0.067
MX 4	0.876	0.875	0.851	0.851	0.875	0.877	0.800	0.800	0.810	0.811	0.721	0.707	0.712	0.693	0.804	0.717
	0.009	0.016	0.026	0.016	0.016	0.015	0.033	0.035	0.027	0.029	0.035	0.061	0.067	0.081	0.077	0.056
MX 5	0.885	0.883	0.851	0.860	0.883	0.878	0.798	0.797	0.815	0.809	0.720	0.687	0.711	0.701	0.806	0.719
	0.011	0.012	0.029	0.015	0.017	0.021	0.043	0.035	0.026	0.032	0.037	0.077	0.081	0.080	0.083	0.062
MX 6	0.884	0.886	0.854	0.855	0.881	0.882	0.807	0.8	0.81	0.815	0.724	0.681	0.707	0.698	0.806	0.717
	0.011	0.013	0.022	0.016	0.018	0.017	0.038	0.035	0.023	0.027	0.042	0.097	0.069	0.078	0.084	0.058
MX 7	0.881	0.885	0.849	0.86	0.878	0.876	0.797	0.796	0.808	0.812	0.725	0.691	0.703	0.689	0.804	0.711
	0.011	0.015	0.019	0.017	0.02	0.022	0.044	0.037	0.028	0.03	0.042	0.076	0.076	0.083	0.083	0.061

Supplementary Table S5. From IBSR to MICCAI 2012 dataset. Average DSC and standard deviation results for **transfer learning**. TL X – transfer learning with X number of target volumes. Structure acronyms are: left thalamus (Tha.L), right thalamus (Tha.R), left caudate (Cau.L), right caudate (Cau.R), left putamen (Put.L), right putamen (Put.R), left pallidum (Pal.L), right pallidum (Pal.R), left hippocampus (Hip.L), right hippocampus (Hip.R), left amygdala (Amy.L), right amygdala (Amy.R), left accumbens (Acc.L), right accumbens (Acc.R) and average value (Avg.)

# imgs	Tha.l	Tha.r	Cau.l	Cau.r	Put.l	Put.r	Pal.l	Pal.r	Hip.l	Hip.r	Amy.l	Amy.r	Acc.l	Acc.r	Avg.	wAvg.
TL 9	0.914	0.912	0.876	0.884	0.914	0.915	0.857	0.862	0.861	0.869	0.808	0.792	0.785	0.763	0.858	0.788
	0.016	0.013	0.094	0.057	0.027	0.024	0.072	0.048	0.018	0.014	0.029	0.040	0.059	0.088	0.070	0.050
TL 11	0.915	0.917	0.879	0.889	0.914	0.915	0.862	0.863	0.858	0.866	0.789	0.793	0.789	0.781	0.859	0.795
	0.015	0.013	0.086	0.050	0.026	0.025	0.056	0.042	0.023	0.017	0.031	0.034	0.055	0.074	0.066	0.044
TL 13	0.915	0.918	0.879	0.887	0.915	0.915	0.863	0.864	0.864	0.870	0.809	0.792	0.796	0.782	0.862	0.800
	0.017	0.014	0.085	0.054	0.027	0.024	0.062	0.042	0.019	0.014	0.028	0.038	0.052	0.080	0.065	0.046
TL 15	0.916	0.918	0.881	0.887	0.916	0.916	0.866	0.870	0.863	0.871	0.814	0.793	0.794	0.785	0.864	0.801
	0.015	0.012	0.084	0.052	0.026	0.023	0.050	0.038	0.019	0.014	0.028	0.038	0.055	0.072	0.063	0.044

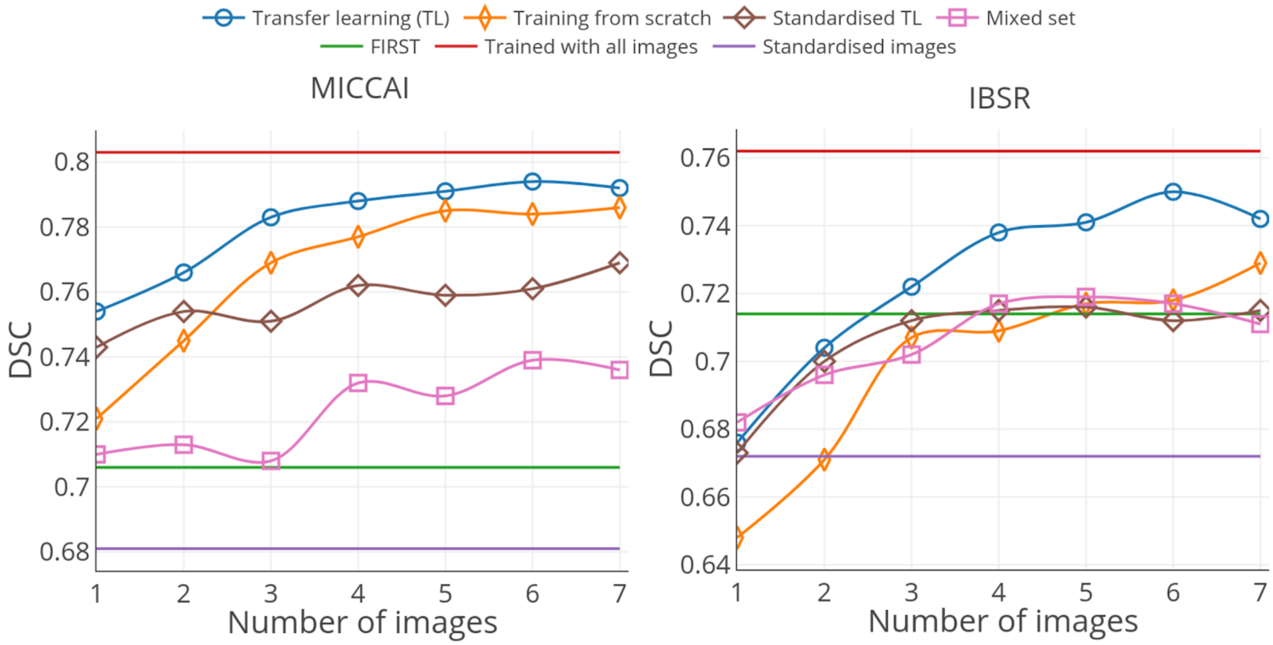

Supplementary Figure S1. Overall **weighted average DSC** results for (**a**) MICCAI 2012 and (**b**) IBSR datasets. The results are shown for seven iterations of transfer learning with original images, training from scratch, transfer learning with standardised images, mixed set training. The horizontal lines correspond to the results of FIRST, full training, and the results of testing standardised images without transfer learning. Training volumes in each iteration for all cases are the same.
